# VISION 2020: The Right to Sight in 7 Years?

**Published:** 2013

**Authors:** Van C. Lansingh, Kristen A. Eckert

**Affiliations:** 1 International Agency for the Prevention of Blindness/VISION 2020 Latin America, Weston, FL 33331, USA; 2 Director International Outreach in the Department of Ophthalmology, Hamilton Eye Institute, University of Tennessee Health Science Center, Memphis, TN 38163, USA; 3 Public Health and Research Consultant (Independent), Fort Collins, CO, 80524, USA

**Keywords:** VISION 2020, The Right to Sight, Prevention of blindness

We applaud the authors and editors of this second issue of the *Medical Hypothesis, Discovery, and Innovation Ophthalmology Journal* in 2013 for presenting new research and innovative ideas on the vast array of issues and diseases related to ocular health. In its scope as a peer-reviewed journal, it covers articles and studies from leading causes of preventable blindness to promising advanced medical therapies. The content focuses on difficult-to-treat diseases that encourage us as eye care professionals to think outside the box, or in our case, slit lamp. We hope that such progressive thinking regarding visual impairment can be extended outside the clinical setting and into the communities, the schools, the nursing facilities, and homes of the patient population. Below we discuss the VISION 2020 program global activities in the prevention of blindness.

This current issue presents a review of promising research in the use stem cell therapy to restore retinitis pigmentosa, which currently is an untreatable condition [1]. Another potentially innovative treatment for a technically complex ocular problem, intracorneal rings for keratoconus, is discussed by Ibrahim et al [2]. In this issue, there are two trials reviewed that explore the use of ozone therapy in dry age-related macular degeneration (AMD) [3], which is a global public health problem and one of the three main causes of blindness [3]. Despite skepticism from the medical community, it is heartening to see the positive results of ozone therapy which has improved the vision of patients almost blind from dry AMD. Dry AMD is a disease that is highly prevalent among the aging population and does not yet have an effective therapy (unlike wet AMD for which we have an increasing arsenal of treatment options). Long-term follow-up and more confirmation studies are required to validate the viability of ozone therapy as a treatment of dry AMD. 

The pathogenesis and treatment intervention of another major, global cause of blindness is also addressed in this issue. Like AMD, glaucoma is a blinding disease that has less effective and costly treatment options, is multifactorial, and is progressive to the point of irreversible blindness. Shahsuvaryan reviewed the role of neuroprotective agents, mainly the potential of calcium channel blockers to mitigate retinal ganglion cell death in patients with glaucoma, and concluded that neuroprotection may be a beneficial adjunctive therapy along with intra-ocular pressure control [4].

Finally, we find it especially worthy to mention the case study presented in this issue on the combination therapy of intravitreal bevacizumb and photocoagulation applied to a 56-year old woman in Argentina who had Eales disease, which provides a patient-centered approach to a difficult and recurring condition [5]. The authors noted that during her follow-up process, optical coherence tomography and angiography were not carried out due to her financial constraints. Fortunately for the patient, this case study ended on a positive note with the disease stabilized, visual acuity improved, and recurrence not evident after four years. Unfortunately, these results are not attainable for many people with visual impairment, because they cannot afford or have limited access to eye care. This particular case illustrates the reality of the so-called “personalized” medical care, but it also unfortunately reveals that despite the relative affluence in a country such as Argentina, there are still barriers to eye health services. What could we then expect for other lesser developed countries? 

Four out of five people lose their sight unnecessarily, even in today’s world, where 80% of blindness can be treated, cured, and/or prevented. The sad reality is that blindness remains a disease of poverty with 90% of people with blindness living in developing countries, where all aspects of health care is often simply not a government funding priority. Although much of the global blindness prevention effort is geared towards the aging population, statistics prove that one child becomes blind every minute. There are 6 million visually-impaired children in the world; 80% of whom live in developing countries. Most of these children will die from other causes within the first year. Improved access to affordable eye care is urgently needed across the globe to protect the millions each year who needlessly go blind. In a responsible global world, efforts need to be made to ensure a future of universal eye health coverage, which should be a fundamental human right.

VISION 2020: *The Right to Sight* (V2020) is a global program that was established in partnership in 1999 by the International Agency for the Prevention of Blindness (IAPB) and the World Health Organization (WHO) with the joint goal to eliminate avoidable blindness by 2020. V2020 is supported worldwide by governments and health ministries, non-governmental organizations (NGOs), eye care professionals, program managers, and industry that together contribute to the planning, development, and implementation of sustainable national eye care programs. These programs are based on the three core pillars of disease control, human resource development and infrastructure development, and the incorporation of the principles of primary health care. National committees for the prevention of blindness have been formed with members from both the private and public sectors in over 80 countries. 

Recent global data from the WHO show that blindness and visual impairment have been reduced globally by 9%, or 26 million people, since 2004 [6]. This is a remarkable achievement in spite of the fact that the age group most affected by visual impairment (>50 years) has increased by 14% in this time frame. Yet, challenges remain in the fight against avoidable blindness, as the WHO estimates that 285 million people worldwide still have visual impairment, and 39 million people are blind. As of 2010, the WHO confirmed that the 3 leading global causes of blindness are cataracts, glaucoma, and AMD [6]. However, diabetes is now a global public health crisis, and diabetic retinopathy, a complication of the disease that leads to blindness, is emerging as a priority of blindness prevention. Uncorrected refractive errors, although quickly solved with an eye examination and eye glasses, are the main cause for moderate to severe visual impairment. 

Again, the reduction in the global cases of blindness and visual impairment is a significant achievement of V2020, which promotes advocacy, carries out relevant research on ophthalmology and public health, and promotes Community Eye Health (CEH) through workshops, training, and partnerships with international, regional, and local ophthalmologic societies. With seven years to go, V2020 has proven to be a successful platform to launch and coordinate blindness prevention activities. However, there is still much work to be done in regions like Latin America, where despite the last decades of increased blindness prevention activities, we still find that more than 60% of the population does not have access to eye care. Although there has been an increase in cataract surgical rates in recent years, the key to successful blindness prevention is coverage. Eye care services coverage is a satisfactory 80% in Latin American cities, but only 10% in rural areas. 

We conducted a recent review of the social determinants of blindness and visual impairment so as to better understand how social determinants based on gender, socioeconomic status, ethnicity, race, living in a specific geographic region, or having a specific health condition influenced the prevalence of visual impairment and blindness [7]. We found these social determinants are indicative of the social inequalities often observed in blindness and visual impairment, which cause the disparities in access and coverage of eye health across certain populations or groups. An example would be the rural, indigenous communities in Latin America, who do not have access to eye care. Our review produced four important findings: (1) women have a higher prevalence of visual impairment and blindness, (2) a higher socioeconomic status was inversely associated with prevalence of blindness or visual impairment, (3) ethnicity and race were associated with visual impairment, and (4) geographic inequalities were observed to be related to income level and living in a rural area [7]. To overcome the great inequality in the provision of eye care and extend coverage to the vulnerable and underserved populations that are affected by these social determinants of eye health, we and supporters of V2020 plan to scale-up regional programs. To do so, we will start by increasing cataract surgical output, increasing efficiency, decreasing costs, and transitioning local eye care programs to become sustainable and systematically integrated into the population’s country health care programs. We can only attain goals such as these with the provision of proper training and comprehensive education to residents and other eye care professionals to better balance the poor geographic distribution of manpower and infrastructure across regions. That way, eye care professionals can work with or establish successful and sustainable programs that maximize impact and service delivery to the globe’s underserved populations and minimize cost to eliminate barriers to eye care.

CEH training is one of the most important V2020 activities and has demonstrated positive impact and results since program inception. In another recent paper [8], we discuss the adoption of CEH training in the residency curriculum of the International Council of Ophthalmology in 2012. CEH training, as a result of V2020 activity, has also been included in regional and national residency curricula and programs, including the Pan American Association of Ophthalmology curriculum and residency programs in Brazil, Chile, and Mexico. In our paper [8], we show how more CEH training is still needed for the future with the number of individuals aged ≥60 years is increasing twice as fast as the number of ophthalmologists, which forebodes greater difficulty in the future as this aging global population will need access to eye care. We advocate for the need to train more ophthalmologists and other eye care professionals to advance the V2020 initiative. At the core of CEH training is the need to change behaviors of ophthalmologists so that they better meet the needs of their local population base and community. In many developing countries, this means the promotion of high volume and cost-effective cataract surgery (where many ophthalmologists currently do not operate or operate on a very limited number of cataracts). A CEH curriculum is based on the principles of public health and is not only about increasing patient-centered approaches of ophthalmologists to eye care. V2020 also offers CEH management courses and a Masters in Science in CEH at the International Centre of Eye Health of the London School of Hygiene and Tropical Medicine. These courses focus on manpower and management training through a systematic, integrated approach to managing comprehensive eye care programs and include the relevant issues of financial planning, fundraising, and human resources. The ultimate goal of these courses is to have able manpower to strengthen regional and national eye care systems through holistic integration of eye care in the general health systems. 

Global and regional data indicate decreasing trends in blindness and visual impairment, but in order for V2020 to be successful in just seven years time, more likeminded eye care professionals working to provide better coverage and accessibility are needed so that no patient is turned away from treatment and becomes needlessly blind. We hope that this journal’s readers will especially consider our message and join us in the fight against avoidable blindness. For further information, please refer to the V2020 website: www.iapb.org/vision-2020. Finally, we extend our gratitude and appreciation to the *Medical Hypothesis, Discovery, and Innovation Journal* for providing us this opportunity to share our research and experience with V2020.

## DISCLOSURE

VC Lansingh is an employee of IAPB/V2020 Latin America; KA Eckert is a consultant to IAPB/V2020 Latin America.
